# Longitudinal hemoglobin trajectories and acute kidney injury in patients undergoing cardiac surgery: a retrospective cohort study

**DOI:** 10.3389/fcvm.2023.1181617

**Published:** 2023-05-17

**Authors:** Shouqiang Zhu, Peng Lu, Zhenran Liu, Shaoyang Li, Peitong Li, Bingdi Wei, Jiayi Li, Yupei Wang

**Affiliations:** ^1^ Department of Anesthesiology and Perioperative Medicine, Xijing Hospital, Fourth Military Medical University, Xi’an, China; ^2^ The First Clinical Medical College, Gansu University of Chinese Medicine, Lanzhou, China; ^3^ Department of Obstetrics and Gynecology, The First Affiliated Hospital of Anhui Medical University, Hefei, China; ^4^ NHC Key Laboratory of Study on Abnormal Gametes and Reproductive Tract (Anhui Medical University), Hefei, China; ^5^ Key Laboratory of Population Health Across Life Cycle (Anhui Medical University), Ministry of Education of the People’s Republic of China, Hefei, China; ^6^ The Second Clinical Medical College of Anhui Medical University, Hefei, China; ^7^ The Third Clinical Medical College of Zhejiang University of Traditional Chinese Medicine, Zhejiang, China; ^8^ School of Public Health, Gansu University of Chinese Medicine, Lanzhou, China; ^9^ The Center for Medical Genetics in Gansu Provincial Maternity and Child-Care Hospital, Gansu Provincial Clinical Research Center for Birth Defects and Rare Diseases, Lanzhou, China

**Keywords:** anemia, acute kidney injury, cardiopulmonary bypass, transfusion, trajectory

## Abstract

**Object:**

The purpose of this study was to describe the longitudinal dynamic hemoglobin trajectories in patients undergoing cardiac surgery and to explore whether they provide a broader perspective in predicting AKI compared to traditional threshold values. Additionally, the interaction of red blood cell transfusion was also investigated.

**Methods:**

The MIMIC-IV database was searched to identify patients undergoing cardiac surgery with cardiopulmonary bypass. Group-based trajectory modeling (GBTM) was used to determine the hemoglobin trajectories in the first 72 h after ICU admission. The correlation between hemoglobin trajectories and AKI was evaluated using multivariable logistic regression and inverse probability of treatment weighting. Receiver operating characteristic (ROC) curves were created in the dataset to further validate previously reported thresholds.

**Results:**

A total of 4,478 eligible patients were included in this study. Three hemoglobin trajectories were identified by GBTM, which were significantly different in the initial hemoglobin level and evolution pattern. Compared to the “the lowest, rising, and then declining” trajectory, patients in the “the highest, declining” and “medium, declining” trajectory groups had significantly lower AKI risk (OR 0.56; 95% CI 0.48, 0.67) and (OR 0.70; 95% CI 0.55, 0.90), respectively. ROC analysis yielded a disappointing result, with an AUC of 0.552, sensitivity of 0.25, and specificity of 0.86 when the hemoglobin threshold was set at 8 g/dl in the entire cohort. In the subgroup analysis of red blood cell transfusion, hemoglobin levels above 10 g/dl predicted higher AKI risk, and there was no correlation between hemoglobin trajectories and AKI in the non-red blood cell transfusion subgroup.

**Conclusion:**

This study identified a hemoglobin trajectory that is associated with an increased risk of AKI after cardiac surgery. It is noteworthy that fixed hemoglobin thresholds should not be applied to all patient types. In patients receiving red blood cell transfusion, maintaining hemoglobin levels above 10 g/dl through transfusion was associated with an increased risk of AKI.

## 1. Introduction

Acute kidney injury (AKI) is a common complication after cardiac surgery, with a reported prevalence between 5% and 42% (1–3). Individuals with severe cardiac surgery-associated acute kidney injury (CS-AKI) were reported to have a three- to eightfold greater risk of mortality and morbidity, resulting in longer hospital and intensive care unit stays and higher healthcare costs (4). Even a mild elevation in creatinine levels (i.e., ≥0.3 mg/dl) can often signal poor outcomes (5). Moreover, patients who experience postoperative AKI and subsequently recover are at a heightened risk of developing long-term chronic kidney disease and end-stage renal disease compared to healthy individuals (6). Regrettably, effective interventions for CS-AKI remain elusive (7), underscoring the importance of identifying modifiable risk factors to mitigate the incidence of AKI among cardiac surgery patients.

Anemia is a common risk factor for acute kidney injury (AKI) in patients undergoing cardiac surgery (8–10). The underlying biological mechanism is complex and multifactorial. One major contributing factor is reduced oxygen delivery to the kidney due to decreased hemoglobin levels, which results in tissue hypoxia, including the kidney. This hypoxia triggers a cascade of events, including the release of vasoconstrictive and pro-inflammatory mediators, which cause renal vasoconstriction and decrease renal blood flow (11). Furthermore, anemia leads to an increase in cardiac output to compensate for reduced oxygen-carrying capacity, and this increased cardiac workload can exacerbate renal hypoperfusion by causing intravascular volume depletion and increasing renal oxygen demand (12, 13).

Perioperative anemia is a critical predictor of CS-AKI (14), and there is currently no established minimum acceptable hemoglobin level during cardiac surgery. While it has been presumed that a hematocrit level of ≥21% or higher during cardiac surgery reduces the incidence of AKI (15), the strength of this recommendation is questionable due to the heterogeneous and dynamic nature of AKI pathophysiology (16). Moreover, previous epidemiological studies examining the relationship between AKI and perioperative anemia have primarily focused on hemoglobin level at a specific time point (17), which may not accurately reflect the dynamic changes in hemoglobin levels during cardiac surgery. A better understanding of hemoglobin level dynamics could enable early identification of patients at risk for CS-AKI and facilitate personalized and targeted therapy.

To address this issue, Group-Based Trajectory Modeling (GBTM), also known as Semiparametric Mixture Model, can be utilized to analyze longitudinal data and explore population heterogeneity (18). GBTM enables the monitoring of changes in hemoglobin levels over time and the identification of different populations with similar longitudinal response patterns (19, 20). Therefore, the primary objective of this study is to describe the dynamic hemoglobin trajectories of patients undergoing cardiac surgery using GBTM and determine whether hemoglobin trajectories can serve as a novel and more valid diagnostic criterion for CS-AKI.

## 2. Method and materials

### 2.1. Sources of data and ethics compliance

Data were extracted from the MIMIC-IV database, which contains detailed information on 76,540 ICU admissions of 53,150 de-identified patients at the Beth Israel Deaconess Medical Center (BIDMC) from 2008 to 2019 (21). This information includes demographic data, laboratory test results, medications, vital signs, nursing notes, and radiology reports, which were obtained from digital electronic health records and hosted by the Laboratory for Computational Physiology at MIT. For privacy considerations, the patient information in this publicly accessible database has been de-identified. The use of the MIMIC database was granted approval by both the BIDMC institutional review board (2001-P-001699/15) and MIT (Approval ID: 10734458). The anonymization of the data permitted us to waive the requirement for informed consenting process. The study followed the Strengthening the Reporting of Observational Studies in Epidemiology (STROBE) guidelines (22).

### 2.2. Population selection criteria

We will include patients who have undergone cardiac surgery with the aid of cardiopulmonary bypass in our analysis. However, we will exclude patients who have been readmitted to the ICU after surgery and only retain their initial admission records. Additionally, patients without measurements of hemoglobin or creatinine will be excluded, as well as those with preoperative creatinine values >4 mg/dl or pre-existing end-stage renal disease.

### 2.3. Variable extraction

The main exposure factor of this study is the longitudinal trajectory pattern of hemoglobin within the first 72 h after cardiac surgery, with the primary outcome being the development of AKI (acute kidney injury) following cardiac surgery. The diagnosis of AKI relies on the KDIGO (Kidney Disease Improving Global Outcomes) diagnostic guidelines, which define AKI as an increase in serum creatinine by more than 0.3 mg/dl or 1.5 times the baseline value. The severity of AKI is also staged according to the KDIGO guidelines, with stage I defined as an increase in serum creatinine by 1.5 times the baseline value or an increase of more than 0.3 mg/dl, stage II as an increase in serum creatinine by 2 times the baseline value, and stage III as an increase in serum creatinine by 3 times the baseline value or serum creatinine greater than 4 mg/dl, or the need for renal replacement therapy after surgery (23). Covariates related to the study include demographic data (age, gender, race, and weight at admission), preoperative comorbidities (diabetes, hypertension, heart failure, and coronary artery disease), laboratory tests (measured within the first 24 h after ICU admission, with the value that best reflects disease severity selected if multiple measurements are taken), treatment-related measures (red blood cell transfusion, surgical method, use of vasoactive drugs, and coronary angiography), and clinical assessments that reflect patient prognosis (length of hospital stay, initial SOFA score, cardiac output, and in-hospital mortality).

Variables with missing proportions exceeding 10% were excluded from our study. The missing values were replaced with the mean or median value (24). The details of the proportion of missing variables can be found in the supplementary material (Supplementary Figure S1).

### 2.4. Statistical methods

Continuous variables that follow a normal distribution are described as mean and standard deviation (SD) and compared between different hemoglobin trajectory patterns using analysis of variance (ANOVA). Non-normally distributed continuous variables are summarized as median and interquartile range (IQR) and compared between different patterns using Wilcoxon rank-sum test or Kruskal-Wallis test. Variable normality was assessed using the Kolmogorov-Smirnov test. Categorical variables were described in terms of frequency (*n*) and proportion (%), and tested using chi-square or Fisher's exact tests.

The longitudinal trajectory of hemoglobin was identified using group-based trajectory modeling (GBTM). The “traj” command in Stata 17 software was used to identify and determine the trajectory. The number and shape of trajectories were determined through a two-stage iterative model fitting process. First, the number of groups was determined by modeling each trajectory group as a high-order shape (i.e., a cube). Then, models with different numbers of groups were compared, starting from one group (no distinct trajectory) up to six groups. After determining the number of groups, the model was run to determine the shape of each trajectory. The Bayesian information criterion (BIC) and Akaike information criterion (AIC) values and Bayes factor were compared to determine the best-fit model. Bayes factor is approximately twice the difference between the Bayes information criterion of the more complex model and that of the simpler model [2 × (Bayes information criterion of more complex model—Bayes information criterion of simpler model)]. A Bayes factor greater than 2 indicates positive evidence in favor of a meaningful change in the Bayes information criterion in support of the more complex model, while a Bayes factor of 10 or greater provides very strong evidence. Additionally, each participant was assigned to a model with an average posterior probability of approximately 70% or higher, indicating a good fit, and models with a membership of greater than 5% in each trajectory group were selected (Supplementary Table S1).

Subsequently, a multivariable logistic regression model was used to estimate the association between the longitudinal trajectory of hemoglobin and the incidence of AKI following cardiac surgery. The model adjusted for variables, including covariates with imbalanced distribution between the AKI and non-AKI groups at baseline. In addition, the following principles were used for variable selection: variables that exhibited clinical or statistical collinearity (Supplementary Table S2) were removed; variables with a univariate *p* value <0.2 or those that showed a change in the effect estimate of >10% after multivariable adjustment were included; and variables with previous literature evidence supporting their association with CS-AKI were also included in the multivariable model. Inverse probability weighting regression adjustment was also used to control for confounding and obtain robust estimates (baseline and clinical characteristics before and after the inverse probability weighting was shown in Supplementary Figure S2). The hemoglobin trajectory allocation (propensity score) model included variables that were imbalanced in the hemoglobin trajectory. A sensitivity analysis according to AKI severity is required, we excluded patients with severe AKI, i.e., those with KDIGO stage III AKI, and subsequently re-modeled the sensitivity analysis cohort using the methods described above. We compared the crude incidence rates of AKI among different hemoglobin trajectory groups and explored whether the adjusted relationships were consistent with the original cohort. The diagnostic test was conducted to explore the utility of previously reported Hb level thresholds in predicting AKI occurrence in our dataset. Specifically, we calculated the sensitivity, specificity, positive predictive value (PPV), negative predictive value (NPV), and area under the curve (AUC) for each threshold using receiver operating characteristic (ROC) analysis. The diagnostic performance of each threshold was evaluated in comparison to our actual AKI diagnosis.

In this study, statistical analysis was performed using R software (version 4.1.2, www.r-project.org) and Stata version 17 (StataCorp LLC, College Station, TX, USA). The significance level was set at *p* < 0.05, indicating statistical significance.

## 3. Results

### 3.1. Study cohort

**Figure 1:**
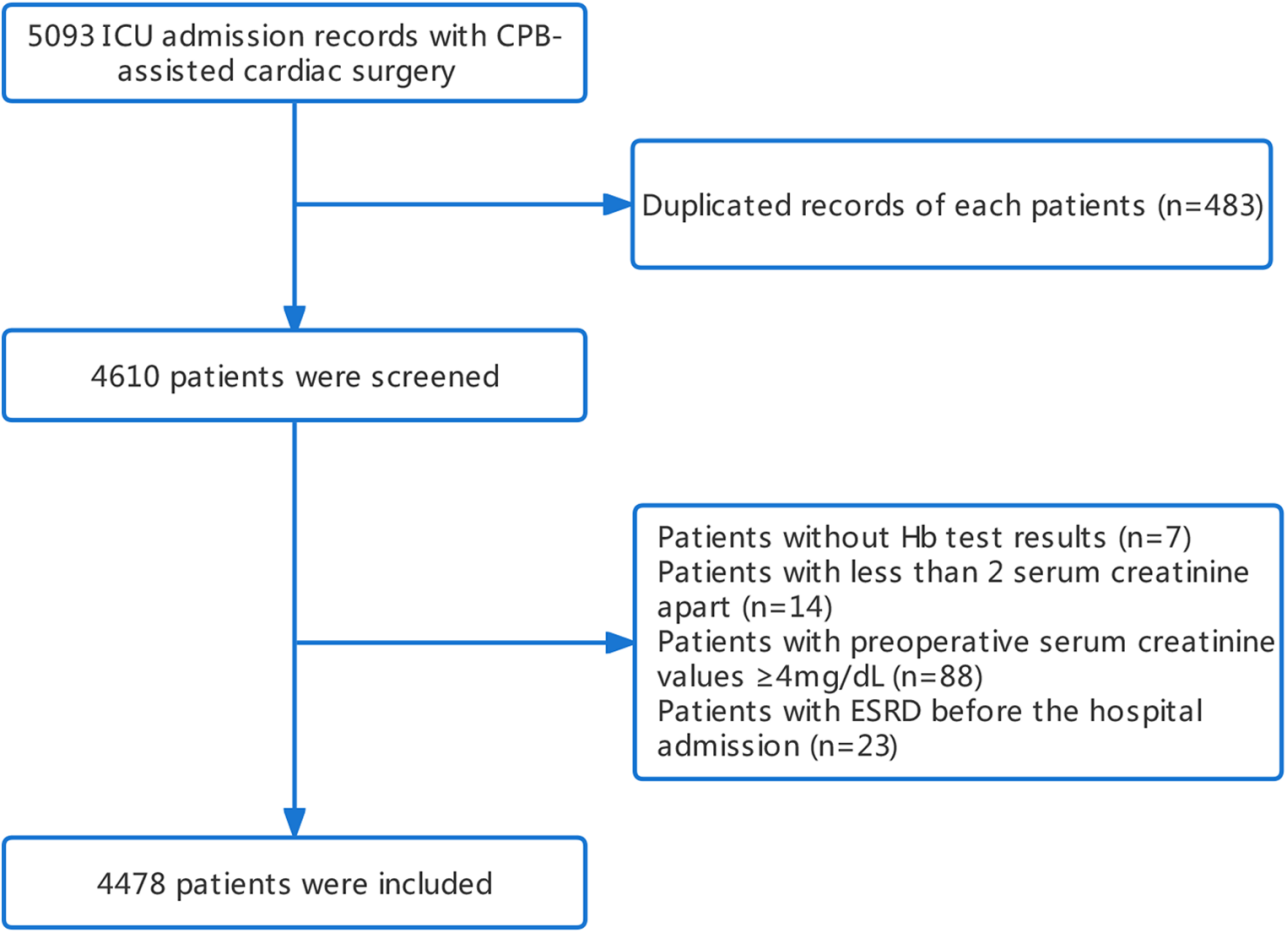
Study flow diagram for analytical sample.

Initially, a total of 5,093 patients who underwent cardiac surgery with cardiopulmonary bypass were identified from the MIMIC-IV database. After applying exclusion criteria, 4,478 patients were ultimately included in the study cohort, among whom 794 (17.73%) were diagnosed with AKI (Figure 1). Overall, the AKI group was older than the non-AKI group (73.0 [65.0; 80.9] vs. 67.7 [60.0; 75.5], *p* < 0.001), and had a lower proportion of male patients (52.4% vs. 73.2%, *p* < 0.001). Compared to the non-AKI group, the AKI group had similar initial (9.30 [8.10; 10.7] vs. 9.90 [8.70; 11.2], *p* < 0.001) and maximum hemoglobin levels (11.4 [10.5; 12.3] vs. 11.5 [10.6; 12.6], *p* < 0.007), although there were statistical differences. A higher percentage of patients in the AKI group received red blood cell transfusion (69.9% vs. 37.6%, *p* < 0.001) and vasopressor support (89.2% vs. 81.6%, *p* < 0.001) (Table 1).

**Table 1:** Comparison of baseline characteristics of the group with and without acute kidney injury.

Variables	Non-AKI group (*N* = 3,684)	AKI group (*N* = 794)	*p*
Age (years, median [IQR])	67.7 [60.0; 75.5]	73.0 [65.0; 80.9]	<0.001
Male, *n* (%)	2,697 (73.2%)	416 (52.4%)	<0.001
Ethnicity, *n* (%)			0.358
African American	120 (3.26%)	33 (4.16%)	
Asian	69 (1.87%)	23 (2.90%)	
Caucasian	2,780 (75.5%)	590 (74.3%)	
Hispanic	122 (3.31%)	25 (3.15%)	
Native American	3 (0.08%)	0 (0.00%)	
Other/Unknown	590 (16.0%)	123 (15.5%)	
Initial weight (kg, median [IQR])	82.2 [72.5; 95.0]	78.8 [66.7; 92.0]	<0.001
Emergency, *n* (%)	1,861 (50.5%)	448 (56.4%)	0.003
Co-morbidities, *n* (%)			
Diabetes	1,110 (30.1%)	278 (35.0%)	0.008
Hypertension	2,346 (63.7%)	471 (59.3%)	0.023
HF	866 (23.5%)	297 (37.4%)	<0.001
CHD	2,694 (73.1%)	531 (66.9%)	<0.001
Biochemical indices, median (IQR)			
Initial WBC (10^9^/L)	9.60 [7.40; 12.8]	10.1 [7.60; 13.3]	0.006
Maximal WBC (10^9^/L)	15.5 [12.6; 19.3]	16.7 [13.5; 21.1]	<0.001
Initial Hb (g/dl)	9.90 [8.70; 11.2]	9.30 [8.10; 10.7]	<0.001
Maximal Hb (g/dl)	11.5 [10.6; 12.6]	11.4 [10.5; 12.3]	0.007
Initial platelet (10^9^/L)	176 [136; 229]	177 [122; 246]	0.594
Minimal platelet (10^9^/L)	120 [99.0; 150]	103 [75.0; 130]	<0.001
Initial creat (mg/dl)	0.90 [0.80; 1.10]	1.00 [0.80; 1.40]	<0.001
Maximal creat (mg/dl)	1.00 [0.90; 1.20]	1.50 [1.10; 2.10]	<0.001
Sodium (mmol/L)	138 [136; 139]	137 [135; 139]	<0.001
Potassium (mmol/L)	4.00 [3.80; 4.30]	4.10 [3.80; 4.30]	0.031
Calcium (mmol/L)	1.16 [1.13; 1.19]	1.15 [1.11; 1.19]	<0.001
Lactate (mmol/L)	1.30 [1.00; 1.70]	1.30 [1.00; 1.80]	0.328
Glucose (mmol/L)	107 [97.0; 126]	110 [97.0; 138]	0.005
Treatment measures			
RBC transfusion, *n* (%)	1,384 (37.6%)	555 (69.9%)	<0.001
Operation, *n* (%)			<0.001
Coronary artery bypass grafting	2,057 (55.8%)	296 (37.3%)	
Operation on valves	707 (19.2%)	212 (26.7%)	
Coronary bypass with valves	448 (12.2%)	171 (21.5%)	
Other	472 (12.8%)	115 (14.5%)	
Vasopressor use, *n* (%)^a^	3,005 (81.6%)	708 (89.2%)	<0.001
Coronary angiography, *n* (%)	1,175 (31.9%)	275 (34.6%)	0.146
Clinical evaluation			
Los hospital (day, median [IQR])	6.65 [5.06; 9.63]	9.26 [6.39; 14.8]	<0.001
LOS ICU (day, median [IQR])	1.40 [1.19; 2.49]	3.59 [2.23; 6.37]	<0.001
Initial SOFA (scores, median [IQR])	2.00 [1.00; 4.00]	3.00 [2.00; 5.00]	<0.001
Maximal SOFA (scores, median [IQR])	5.00 [4.00; 7.00]	7.00 [5.00; 10.0]	<0.001
Cardiac output (L/min, median [IQR])^b^	4.50 [3.60; 5.50] (*n* = 2,391)	3.90 [3.10; 4.97] (*n* = 633)	<0.001
Acute posthemorrhagic anemia, *n* (%)	469 (12.7%)	209 (26.3%)	<0.001
In-hospital death, *n* (%)	20 (0.54%)	47 (5.92%)	<0.001
AKI I, *n* (%)	0 (0.00%)	537 (67.6%)	
AKI II, *n* (%)	0 (0.00%)	169 (21.3%)	
AKI III, *n* (%)	0 (0.00%)	88 (11.1%)	

AKI, acute kidney injury; HF, heart failure; CHD, coronary heart disease; WBC, white blood cells; ICU, intensive care unit; Hb, hemoglobin; IQR, interquartile range; LOS, length of stay; RBC, red blood cell; SOFA, Sequential Organ Failure Assessment.

^a^ Vasopressor use including dobutamine, dopamine, epinephrine, norepinephrine phenylephrine and asopressin.

^b^ Data on cardiac output represented in the present study include only participants with no missing data.

### 3.2. Characterization of hemoglobin count trajectories

Our model identified three distinct longitudinal trajectories of hemoglobin levels in Supplementary Table S1, with average posterior probabilities (AvePP) greater than 0.8 for each group and population proportions greater than 5%. Traj-1 (*n* = 1,986, 44.35%) had the lowest initial hemoglobin levels (approximately 8.5 g/dl), slowly increasing to nearly 10 g/dl before gradually decreasing to 9 g/dl. Traj-3 had the highest initial hemoglobin levels, slowly decreasing to nearly 11.5 g/dl (*n* = 538, 12.01%). Traj-2 (*n* = 1,954, 43.64%) had initial hemoglobin levels between Traj-1 and Traj-3, slowly decreasing to nearly 9.5 g/dl (Figure 3). Traj-1 had a significantly higher proportion of patients receiving red blood cell transfusions compared to the other two groups (68.1% vs. 28.6%, 5.2%), and baseline characteristics differed among the trajectory groups as shown in Table 2.

**Figure 2:**
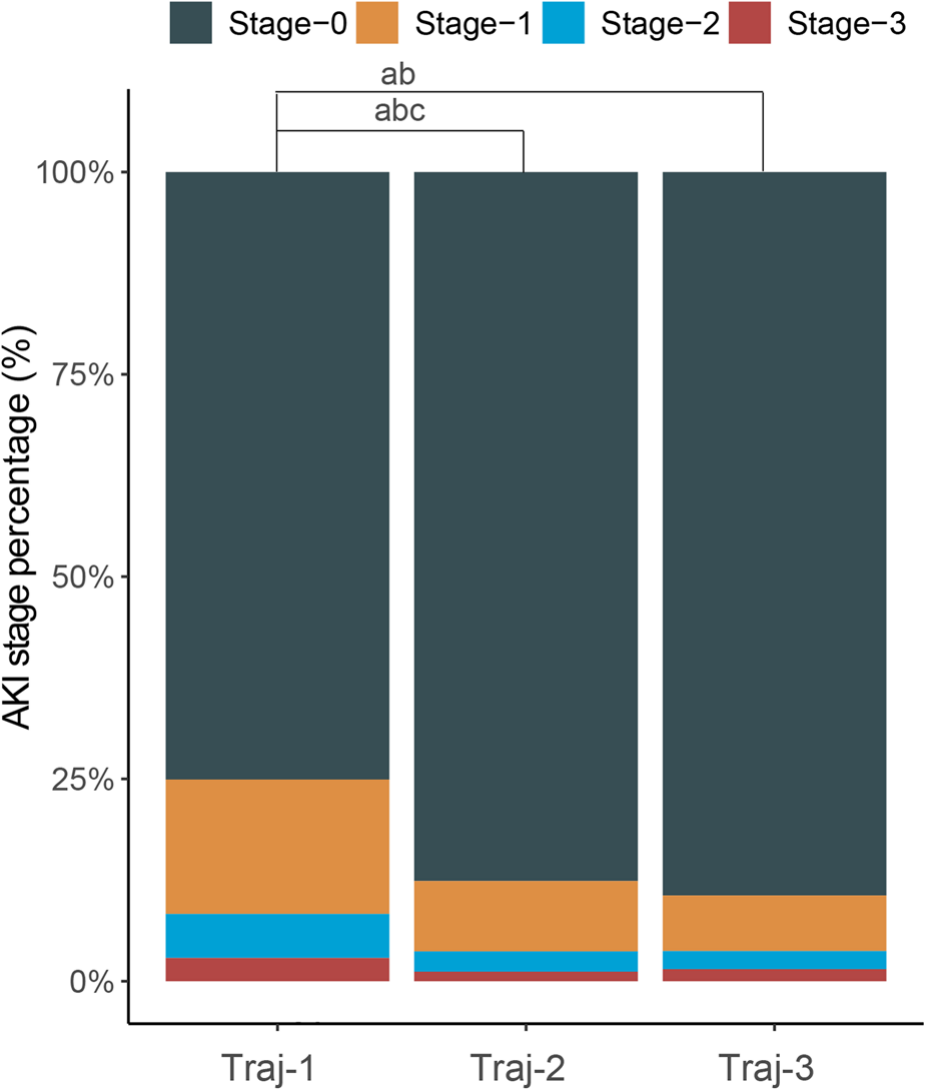
Stacked column chart with the percentage of AKI in stage 0–3 of each hemoglobin trajectories group. a: Significant statictical difference between the Traj-1 and Traj-2/3 in percentage of AKI stage-1; b: significant statictical difference between the Traj-1 and Traj-2/3 in percentage of AKI stage-2; c: significant statictical difference between the Traj-1 and Traj-2/3 in percentage of AKI stage-3.

**Figure 3:**
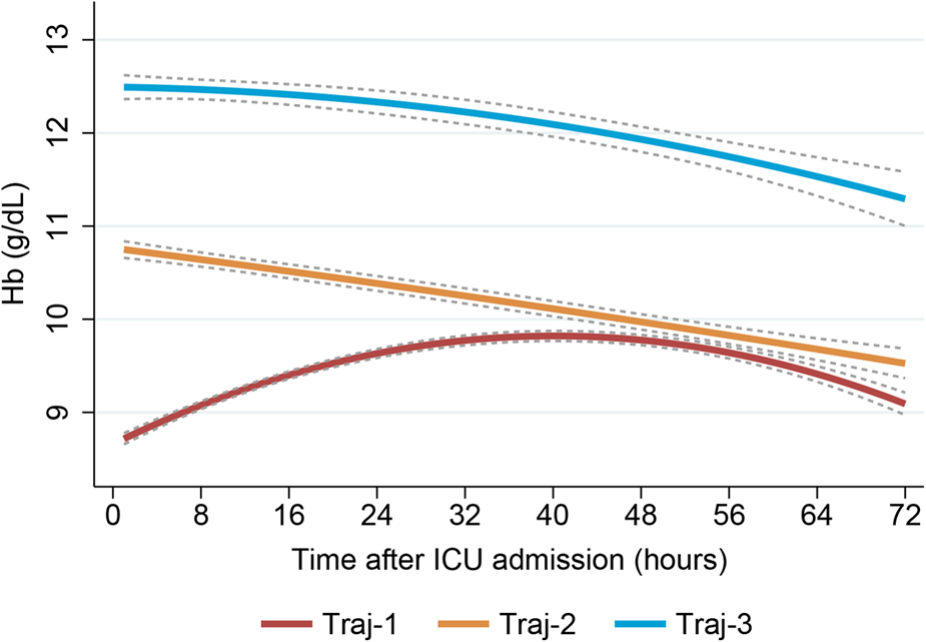
Trajectory plot of patients with three dynamic hemoglobin trajectory patterns.

**Table 2:** Crude comparison within hemgobin trajectories group.

	Traj-1 group (*N* = 1,986)	Traj-2 group (*N* = 1,954)	Traj-3 group (*N* = 538)	*p*
Initial Hb (g/dl)	8.50 [7.70; 9.40]	10.4 [9.60; 11.3]	12.3 [11.4; 13.5]	<0.001
Maximal Hb (g/dl)	10.5 [10.0; 11.1]	12.0 [11.3; 12.6]	13.6 [13.1; 14.3]	<0.001
Initial platelet (10^9^/L)	178 [132; 239]	175 [135; 225]	176 [138; 227]	0.425
Minimal platelet (10^9^/L)	112 [87.0; 143]	119 [97.0; 149]	124 [100; 153]	<0.001
Initial creat (mg/dl)	0.90 [0.80; 1.20]	0.90 [0.80; 1.10]	0.90 [0.80; 1.10]	0.002
Maximal creat (mg/dl)	1.10 [0.90; 1.50]	1.00 [0.90; 1.20]	1.00 [0.90; 1.30]	<0.001
RBC transfusion, *n* (%)	1,353 (68.1%)	558 (28.6%)	28 (5.20%)	<0.001
Operation, *n* (%)				<0.001
Coronary artery bypass grafting	904 (45.5%)	1,112 (56.9%)	337 (62.6%)	
Operation on valves	471 (23.7%)	360 (18.4%)	88 (16.4%)	
Coronary bypass with valves	347 (17.5%)	233 (11.9%)	39 (7.25%)	
Other	264 (13.3%)	249 (12.7%)	74 (13.8%)	
Vasopressor use, *n* (%)^a^	1,732 (87.2%)	1,614 (82.6%)	367 (68.2%)	<0.001
Coronary angiography, *n* (%)	627 (31.6%)	595 (30.5%)	228 (42.4%)	<0.001
Los hospital (day, median [IQR])	7.89 [5.34; 11.5]	6.27 [5.00; 8.89]	6.97 [5.14; 10.3]	<0.001
Los ICU (day, median [IQR])	2.18 [1.29; 3.54]	1.44 [1.19; 2.89]	1.43 [1.15; 2.79]	<0.001
Initial SOFA (scores, median [IQR])	3.00 [1.00; 5.00]	2.00 [1.00; 4.00]	2.00 [0.00; 4.00]	<0.001
Maximal SOFA (scores, median [IQR])	6.00 [4.00; 8.00]	5.00 [4.00; 7.00]	5.00 [3.00; 7.00]	<0.001
Cardiac output (L/min, median [IQR])^b^	4.12 [3.30; 5.10] (*n* = 1,518)	4.60 [3.60; 5.58] (*n* = 1,249)	5.28 [4.14; 6.01] (*n* = 257)	<0.001
Acute posthemorrhagic anemia, *n* (%)	398 (20.0%)	227 (11.6%)	53 (9.85%)	<0.001
In-hospital death, *n* (%)	43 (2.17%)	19 (0.97%)	5 (0.93%)	0.004
AKI Stage, *n* (%)				<0.001
I	330 (16.6%)	170 (8.70%)	37 (6.88%)	
II	108 (5.44%)	49 (2.51%)	12 (2.23%)	
III	57 (2.87%)	23 (1.18%)	8 (1.49%)	

AKI, acute kidney injury; ICU, intensive care unit; Hb, hemoglobin; IQR, interquartile range; LOS, length of stay; RBC, red blood cell; SOFA, Sequential Organ Failure Assessment.

^a^ Vasopressor use including dobutamine, dopamine, epinephrine, norepinephrine phenylephrine and asopressin.

^b^ Data on cardiac output represented in the present study include only participants with no missing data.

### 3.3. Hemoglobin trajectories and CS-AKI

**Figure 5:**
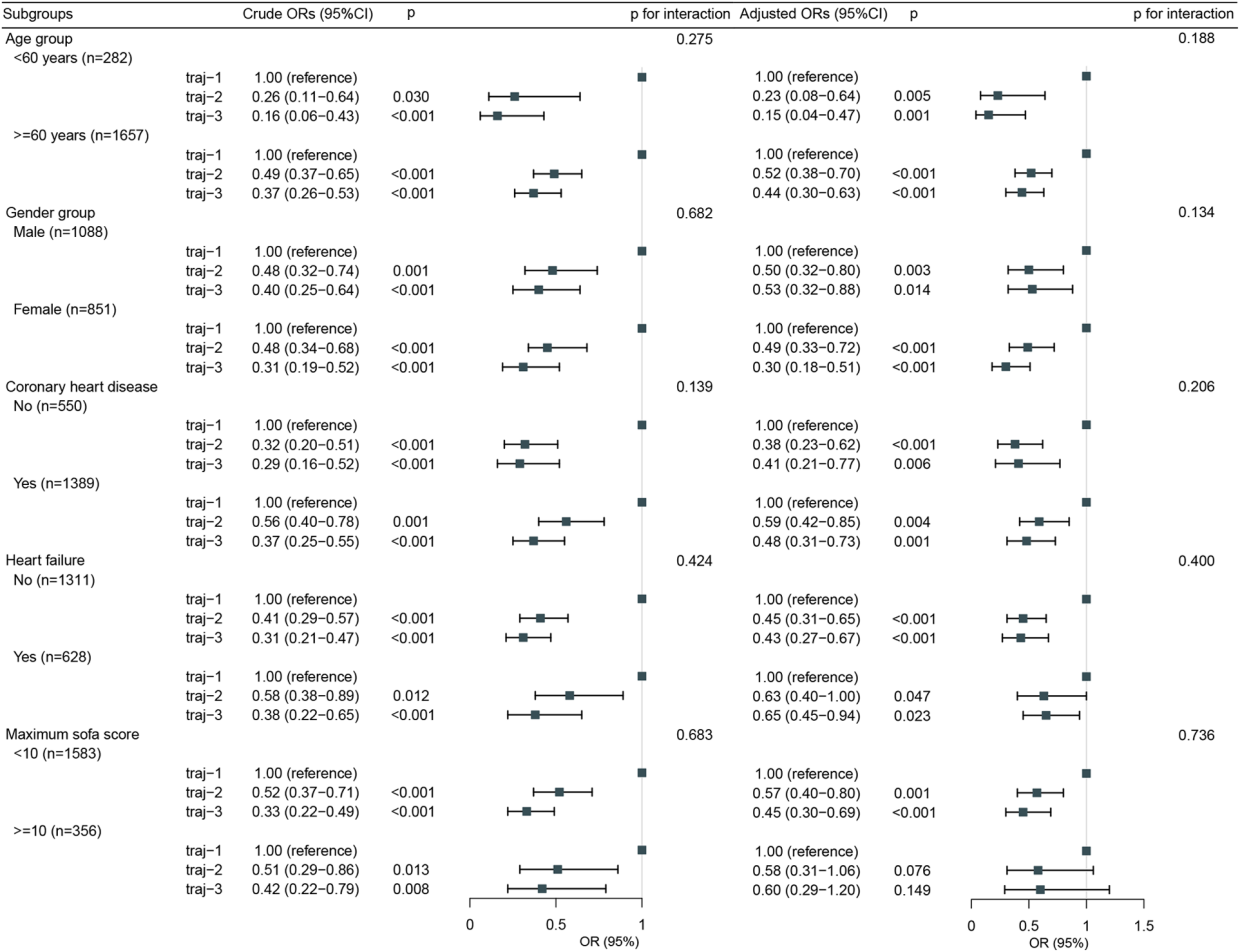
Subgroup analysis for the association of hemglobin trajectories with risk of acute kidney injury.

The overall incidence of AKI in Traj-1 group was higher than that in Traj-2 and Traj-3 groups, and the incidence rates of AKI stages I, II, and III were higher in Traj-1 group than those in Traj-2 group. The incidence rates of AKI stages I and II in Traj-1 group were also higher than those in Traj-3 group (Figure 2). After adjusting for the full model, the AKI risk in Traj-1 group was higher than that in Traj-2 group (OR = 0.61, 95% CI: 0.51–0.73, *p* < 0.001) and Traj-3 group (OR = 0.62, 95% CI: 0.45–0.85, *p* = 0.004). The results remained robust after inverse probability weighting, with the AKI risk in Traj-1 group being higher than that in Traj-2 group (OR = 0.56, 95% CI: 0.48–0.67, *p* < 0.001) and Traj-3 group (OR = 0.70, 95% CI: 0.55–0.90, *p* = 0.007) (Table 3). No heterogeneity was found in the subgroup analysis, and the results remained robust (Figure 5).

**Table 3:** Association of hemoglobin trajectories with risk of AKI in different regression models.

Model	Cluster	Original cohort (*n* = 4,478)			RBC transfusion subgroup (*n* = 1,939)			Non-RBC transfusion subgroup (*n* = 2,539)		
		OR	95% CI	*p*	OR	95% CI	*p*	OR	95% CI	*p*
Unadjusted Model	Traj-1	Reference			Reference			Reference		
	Traj-2	0.43	(0.36–0.50)	<0.001	0.46	(0.35–0.60)	<0.001	0.78	(0.59–1.03)	0.079
	Traj-3	0.36	(0.26–0.47)	<0.001	0.33	(0.24–0.45)	<0.001	0.84	(0.46–1.44)	0.551
Model_1_	Traj-1	Reference			Reference			Reference		
	Traj-2	0.55	(0.46–0.66)	<0.001	0.48	(0.36–0.63)	<0.001	1.04	(0.77–1.40)	0.809
	Traj-3	0.55	(0.40–0.75)	<0.001	0.38	(0.27–0.54)	<0.001	1.21	(0.64–2.13)	0.535
Model_2_	Traj-1	Reference			Reference			Reference		
	Traj-2	0.57	(0.48–0.68)	<0.001	0.49	(0.37–0.65)	<0.001	1.06	(0.79–1.43)	0.697
	Traj-3	0.56	(0.41–0.76)	<0.001	0.41	(0.29–0.58)	<0.001	1.12	(0.60–1.99)	0.705
Model_3_	Traj-1	Reference			Reference			Reference		
	Traj-2	0.58	(0.48–0.69)	<0.001	0.51	(0.38–0.67)	<0.001	1.04	(0.77–1.40)	0.821
	Traj-3	0.54	(0.39–0.74)	<0.001	0.43	(0.30–0.61)	<0.001	1.04	(0.55–1.85)	0.906
Model_4_	Traj-1	Reference			Reference			Reference		
	Traj-2	0.61	(0.51–0.73)	<0.001	0.51	(0.38–0.68)	<0.001	1.09	(0.80–1.48)	0.578
	Traj-3	0.62	(0.45–0.85)	0.004	0.44	(0.31–0.62)	<0.001	1.15	(0.60–2.08)	0.659
Model_IPW_	Traj-1	Reference			Reference			Reference		
	Traj-2	0.56	(0.48–0.67)	<0.001	0.55	(0.41–0.73)	<0.001	1.09	(0.82–1.46)	0.545
	Traj-3	0.70	(0.55–0.90)	0.007	0.45	(0.33–0.63)	<0.001	1.69	(0.96–2.85)	0.570

AKI, acute kidney injury; RBC, red blood cell. IPW, inverse probability of treatment weighting. Model_1_: adjusted for age, gender, initial weight and emergency. Model_2_: additionally adjusted for diabetes, hypertension, hear failure and coronary hear disease upon Model_1_. Model_3_: additionally adjusted for initial white blood cells, serum sodium, serum potassium, serum calcium, serum lactate and serum glucose upon Model_2_. Model_4_: additionally adjusted for vasopressor use, coronary angiography and operation upon Model_3_. Model_IPW_: adjusted for all aforementioned covariates using the IPW method.

### 3.4. Hemoglobin trajectories, red blood cell (RBC) transfusion and CS-AKI

**Figure 4:**
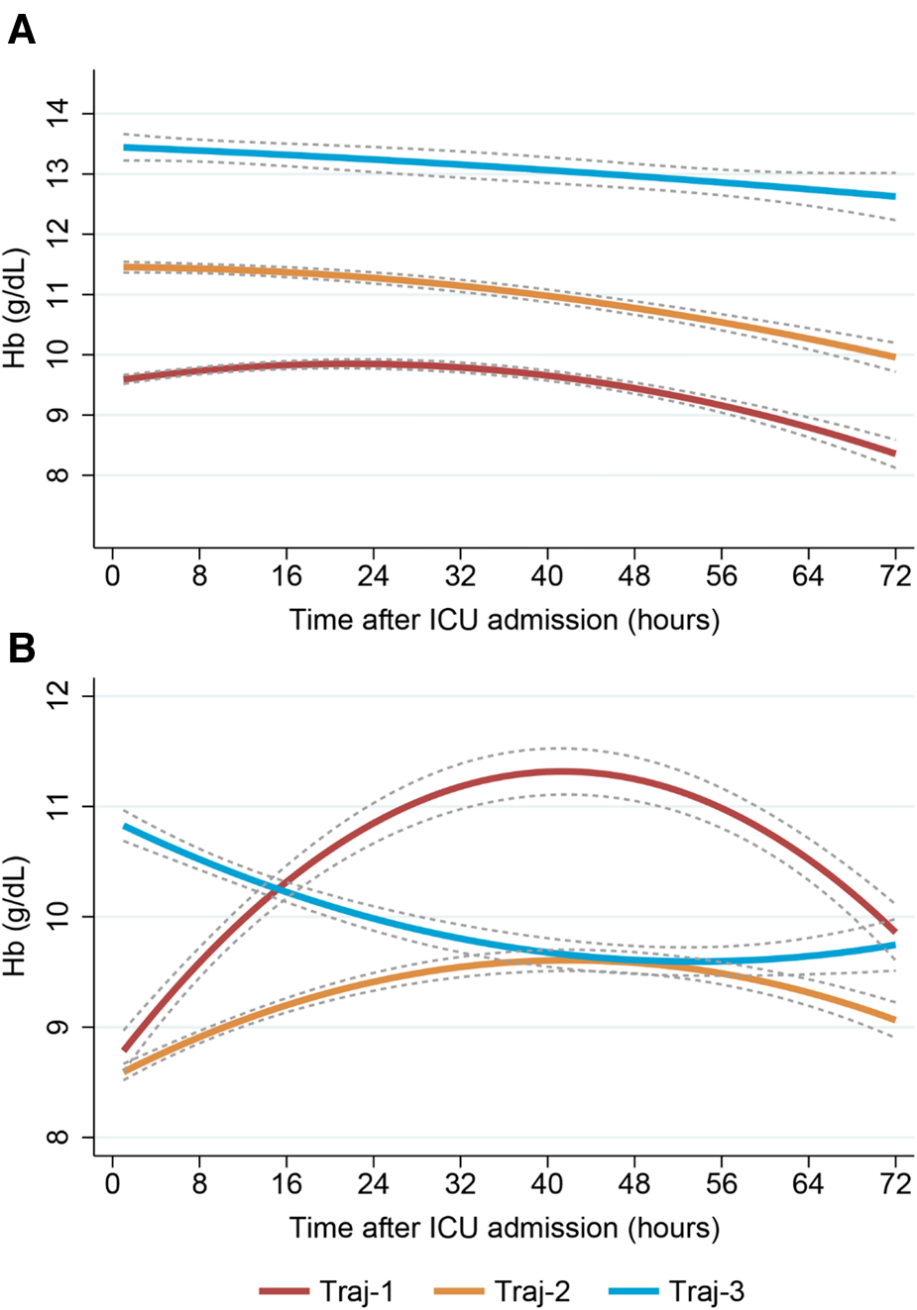
Dynamic hemoglobin trajectory patterns with and without RBC transfusion. (Plot **A**) Non-RBC transfusion group; (Plot **B**) RBC transfusion group. RBC, red blood cell.

After cardiac surgery, red blood cell transfusion can cause an increase in hemoglobin levels. In our study, we found that the Traj-1 group had a significantly higher proportion of patients receiving red blood cell transfusions compared to the other two trajectory groups. Therefore, we conducted a subgroup analysis on red blood cell transfusions. In the non-red blood cell transfusion subgroup, all three hemoglobin trajectory groups showed a consistent decreasing trend, despite differences in initial hemoglobin levels (Figure 4A). In the red blood cell transfusion subgroup, the Traj-1 group had a low initial hemoglobin level (close to 9 g/dl), which increased to 11 mg/dl before decreasing to nearly 10 g/dl. The Traj-2 group had a low initial hemoglobin level (close to 8.5 g/dl), which slowly increased before decreasing to 9 mg/dl. The Traj-3 group had an initial hemoglobin level of 11 g/dl, which decreased to 9.5 g/dl (Figure 4B). After adjusting for the entire model and inverse probability of treatment weighting, we found that in the non-red blood cell transfusion subgroup, there was no correlation between hemoglobin trajectory and the risk of AKI. However, in the red blood cell transfusion subgroup, the Traj-1 group still had a higher risk of AKI compared to the other two trajectory groups (Table 3).

### 3.5. Validation of previous hemoglobin level threshold

We conducted a diagnostic test for hemoglobin level thresholds on the original queue, red blood cell transfusion subgroup, and non-red blood cell transfusion subgroup. The ROC results showed that the AUC (area under the curve) was not higher than 0.6, indicating that a “fixed and universal hemoglobin threshold” approach should be questioned when the thresholds were set at 8 g/dl, 10 dl, and 12 dl (Supplementary Table S3). Perhaps, focusing on the evolution trajectory of hemoglobin may be more appropriate than absolute values.

### 3.6. Sensitivity analysis

After excluding AKI-III patients, we re-modeled and identified a hemoglobin trajectory plot that closely matched the number and evolution pattern of the original queue trajectory (Supplementary Figure S3). We roughly compared three hemoglobin trajectory groups (Supplementary Table S4) and explored the adjusted relationships (Supplementary Table S5), which yielded results consistent with the original queue.

## 4. Discussion

Perioperative anemia has been reported as a risk predictor for postoperative acute kidney injury (AKI) in cardiac surgery. However, static and unchanging hemoglobin levels at a specific time point may not reflect the heterogeneity seen in real-world clinical practice. In our study, we identified three significantly different hemoglobin trajectory patterns using GBTM. The group with the trajectory pattern of “lowest, rising, and then declining” had a significantly higher risk of AKI compared to those in the “highest, declining” and “moderate, declining” trajectory groups. This relationship was validated in multivariable regression and inverse probability weighting models. Additionally, we found that red blood cell transfusion played an interactive role, with the AKI risk being higher in the subgroup with the trajectory pattern of “lowest, rising by more than 10 g/dl, and then declining” among those receiving red blood cell transfusions. However, among those not receiving red blood cell transfusions, three trajectory groups with different initial hemoglobin levels but similar declining trends did not show any correlation with the risk of AKI. Interestingly, our study showed that the previous threshold did not demonstrate good predictive performance in our dataset. Hemoglobin elevation is harmful to the kidneys, and correcting anemia and increasing hemoglobin levels may lead to kidney overcompensation, increasing glomerular filtration pressure and vasoconstriction, which can result in glomerular injury.

Perioperative anemia is common in cardiac surgery and multiple studies have shown a relationship between anemia or low hemoglobin levels and postoperative AKI in cardiac surgery. A retrospective analysis of 1,047 patients undergoing coronary artery bypass graft surgery by Luca and colleagues found that preoperative anemia was an independent risk factor for postoperative AKI and there was no dose-response relationship between the severity of anemia and acute kidney injury (25). However, in a cohort of 920 patients undergoing cardiac surgery with cardiopulmonary bypass, researchers found that the incidence of AKI increased significantly when hemoglobin levels were extremely low (less than 25th percentile) and receiving red blood cell transfusions when hemoglobin concentration was above 8 g/dl also increased the incidence of AKI (26). A study of 1,360 CPB patients found that the lowest hemoglobin level (rather than preoperative anemia) was an independent risk factor for adverse outcomes (27).

However, these studies have the following limitations. Firstly, previous studies have used different thresholds for defining anemia, which has led to inconsistency and difficulty in comparability of results. For example, a retrospective study involving 1,360 cardiac surgery patients defined anemia as preoperative hemoglobin levels below 8 g/dl (27), while a prospective cohort study involving 1,047 patients defined anemia as preoperative hemoglobin levels below 13 g/dl (male) or 12 g/dl (female) (25). These different definitions may have different impacts on the results. Secondly, most studies have used a single preoperative or postoperative measurement of hemoglobin to determine anemia, ignoring the temporal changes in anemia. Or only preoperative hemoglobin levels were used to determine anemia, without considering changes in postoperative hemoglobin levels. This may not reflect the impact of perioperative changes in hemoglobin levels on AKI. Finally, although some studies have explored the relationship between anemia and postoperative AKI in cardiac surgery, the sample sizes of these studies are insufficient, resulting in weak statistical power.

Using group-based trajectory modeling, we identified three distinct trajectories of hemoglobin levels, with the trajectory-1 (traj-1) group showing a significantly higher risk of AKI compared to the other two groups. The traj-1 group had lower initial hemoglobin levels, which reflected the severity of anemia, followed by an increase due to red blood cell transfusion. In our study, a significantly higher proportion of patients in the traj-1 group received red blood cell transfusions compared to the other two groups (68.1% vs. 28.6%, 5.2%). Red blood cell transfusion is known to play a significant role in the development of AKI after cardiac surgery. Transfusion can cause vasoconstriction, decreased oxygen delivery, and inadequate microcirculatory perfusion, leading to renal dysfunction (28, 29). Moreover, transfused blood may contain pathogenic agents such as bacteria and viruses, increasing the risk of postoperative infection and subsequent development of AKI (29, 30).

Therefore, in order to make our research more applicable to decision-making in real-world clinical settings, we performed a subgroup analysis on the use of blood transfusions. In the non-red blood cell transfusion subgroup, we identified three trajectories with consistent initial hemoglobin levels but subsequent decreases. Further analysis revealed that the incidence of AKI did not differ significantly among the different hemoglobin trajectory groups. Even after adjusting for confounding factors and conducting IPW analysis, we did not find any correlation between hemoglobin trajectories and AKI, which contradicts previous conclusions that anemia is associated with an increased risk of AKI (27, 31, 32). This can be explained by several factors. First, previous studies have focused more on the correlation between preoperative anemia and postoperative AKI, and there is little literature on the correlation between postoperative anemia and AKI. A retrospective observational study including 6,130 patients who underwent coronary artery bypass surgery showed that preoperative anemia and preoperative anemia combined with postoperative anemia were associated with AKI and mortality rates after coronary artery bypass surgery, but no correlation was found between postoperative anemia and AKI (8). Second, most studies have not taken into account the impact of red blood cell transfusions on the results, especially postoperative red blood cell transfusions. Mixing patients who received red blood cell transfusions with those who did not can significantly bias the study results. Finally, in the non-red blood cell transfusion subgroup we studied, the lowest hemoglobin level was >8 g/dl, which provides sufficient oxygen to renal tissue. A higher hemoglobin level does not improve renal tissue oxygenation and therefore does not reduce the risk of AKI. A sub-study of a multicenter RCT exploring the hemoglobin threshold for receiving red blood cell transfusions showed that the incidence of AKI did not decrease when a restrictive transfusion strategy was implemented (limiting transfusions) and when the hemoglobin concentration was maintained above 8.5 g/dl compared to a strategy where transfusions were only performed when the hemoglobin threshold was below 7.5 g/dl (33).

In the subgroup of red blood cell transfusion, we also identified three distinct trajectories. The AKI risk in the traj-1 group, which showed a significant increase in hemoglobin levels followed by a subsequent decrease, was higher than that in the group with initially low hemoglobin levels that slowly increased and the group with initially normal hemoglobin levels that subsequently decreased. This suggests that the risk of AKI significantly increases when hemoglobin levels rise to 10 g/dl after red blood cell transfusion. In our study, patients in the traj-1 group of the red blood cell transfusion subgroup received a greater volume of red blood cell transfusions than those in the other trajectory groups (Table 4).

**Table 4:** Crude comparison within hemgobin trajectories group with or without RBC transfusion.

	Non-RBC transfusion subgroup				RBC transfusion subgroup			
	Traj-1 group	Traj-2 group	Traj-3 group	*p*	Traj-1 group	Traj-2 group	Traj-3 group	*p*
	*N* = 1,128	*N* = 1,245	*N* = 166		*N* = 280	*N* = 1,193	*N* = 466	
Initial Hb (g/dl)	11.2 [10.4; 12.1]	9.15 [8.40; 9.90]	13.5 [12.5; 14.7]	<0.001	8.70 [7.80; 10.4]	8.50 [7.60; 9.50]	10.4 [9.60; 11.4]	<0.001
Maximal Hb (g/dl)	12.6 [12.0; 13.2]	10.8 [10.2; 11.4]	14.6 [13.9; 15.2]	<0.001	11.9 [11.4; 12.5]	10.5 [10.0; 11.0]	12.0 [11.3; 12.8]	<0.001
Initial platelet (10^9^/L)	177 [141; 223]	181 [141; 238]	178 [133; 246]	0.230	161 [119; 230]	175 [128; 239]	166 [125; 227]	0.054
Minimal platelet (10^9^/L)	128 [103; 155]	125 [102; 155]	114 [96.2; 153]	0.025	94.0 [63.0; 121]	109 [85.0; 137]	104 [80.0; 127]	<0.001
Initial creat (mg/dl)	0.90 [0.80; 1.10]	0.90 [0.70; 1.10]	0.90 [0.80; 1.10]	0.210	0.90 [0.80; 1.30]	1.00 [0.80; 1.30]	0.90 [0.80; 1.10]	0.031
Maximal creat (mg/dl)	1.00 [0.90; 1.20]	1.00 [0.80; 1.30]	1.10 [0.90; 1.30]	0.043	1.20 [0.90; 1.70]	1.20 [0.90; 1.60]	1.10 [0.90; 1.40]	0.001
Operation, *n* (%)				<0.001				<0.001
Coronary artery bypass grafting	609 (54.0%)	779 (62.6%)	107 (64.5%)		95 (33.9%)	523 (43.8%)	240 (51.5%)	
Operation on valves	240 (21.3%)	213 (17.1%)	29 (17.5%)		80 (28.6%)	278 (23.3%)	79 (17.0%)	
Coronary bypass with valves	130 (11.5%)	95 (7.63%)	9 (5.42%)		63 (22.5%)	241 (20.2%)	81 (17.4%)	
Other	149 (13.2%)	158 (12.7%)	21 (12.7%)		42 (15.0%)	151 (12.7%)	66 (14.2%)	
Vasopressor use, *n* (%)^a^	904 (80.1%)	944 (75.8%)	94 (56.6%)	<0.001	255 (91.1%)	1,078 (90.4%)	438 (94.0%)	0.060
Coronary angiography, *n* (%)	329 (29.2%)	426 (34.2%)	68 (41.0%)	0.002	86 (30.7%)	389 (32.6%)	152 (32.6%)	0.081
Los hospital (day, median [IQR])	6.42 [4.93; 9.17]	6.06 [4.81; 8.31]	7.17 [5.31; 12.6]	<0.001	8.99 [6.35; 13.9]	8.23 [5.97; 11.9]	6.94 [5.31; 10.1]	<0.001
Los icu (day, median [IQR])	1.33 [1.17; 2.18]	1.29 [1.12; 2.20]	1.53 [1.21; 3.05]	<0.001	3.36 [2.21; 6.22]	2.35 [1.38; 4.19]	2.90 [2.00; 4.20]	<0.001
Initial sofa (scores, median [IQR])	2.00 [1.00; 4.00]	2.00 [1.00; 4.00]	1.00 [0.00; 3.00]	<0.001	3.00 [2.00; 6.00]	3.00 [1.00; 5.00]	2.00 [1.00; 4.00]	<0.001
Maximal sofa (scores, median [IQR])	5.00 [4.00; 7.00]	5.00 [4.00; 7.00]	5.00 [3.00; 7.00]	0.051	8.00 [6.00; 10.0]	6.00 [5.00; 8.00]	6.50 [5.00; 9.00]	<0.001
Cardiac output (L/min, median [IQR])^b^	4.60 [3.70; 5.40] (*n* = 713)	5.10 [4.14; 5.96] (*n* = 663)	5.55 [4.58; 6.36] (*n* = 75)	<0.001	3.40 [2.80; 4.30] (*n* = 253)	4.10 [3.30; 5.10] (*n* = 962)	4.20 [3.20; 5.09] (*n* = 358)	<0.001
In-hospital death, *n* (%)	8 (0.71%)	5 (0.40%)	3 (1.81%)	0.084	15 (5.36%)	27 (2.26%)	9 (1.93%)	0.008
AKI Stage, *n* (%)				0.221				<0.001
I	92 (8.16%)	79 (6.35%)	8 (4.82%)		83 (29.6%)	213 (17.9%)	62 (13.3%)	
II	22 (1.95%)	17 (1.37%)	5 (3.01%)		28 (10.0%)	74 (6.20%)	23 (4.94%)	
III	5 (0.44%)	9 (0.72%)	2 (1.20%)		16 (5.71%)	41 (3.44%)	15 (3.22%)	
RBC transfusion (ml, median [IQR])					1,088 [700; 1,750]	700 [350; 1,112]	700 [350; 1,050]	<0.001

AKI, acute kidney injury; ICU, intensive care unit; Hb, hemoglobin; IQR, interquartile range; LOS, length of stay; RBC, red blood cell; SOFA, Sequential Organ Failure Assessment.

^a^ Vasopressor use including dobutamine, dopamine, epinephrine, norepinephrine phenylephrine and asopressin.

^b^ Data on cardiac output represented in the present study include only participants with no missing data.

The increase in hemoglobin levels after red blood cell transfusion is due to the large amount of hemoglobin contained in the transfused red blood cells. Transfusion of red blood cells and hemolysis caused by cardiopulmonary bypass can lead to an increase in the release of free hemoglobin. The free hemoglobin is filtered through the glomerulus and then reabsorbed and metabolized, with the resulting metabolites being excreted by the kidneys. Therefore, high concentrations of hemoglobin can increase the burden on the kidneys, leading to impaired kidney function (29). Additionally, red blood cell transfusion can cause inflammatory and oxidative stress reactions. The transfused blood may contain pathogenic substances such as bacteria and viruses, which can trigger an immune system response and generate a large amount of inflammatory mediators. These inflammatory mediators can promote kidney tissue inflammation and contribute to impaired kidney function (30). Furthermore, red blood cell transfusion may cause oxidative stress reactions, resulting in the production of large amounts of free radicals and oxidants within cells, which can damage kidney tissue and exacerbate the occurrence of acute kidney injury (34). Finally, red blood cell transfusion may lead to changes in hemodynamics. Transfusion of blood can cause vasoconstriction and inadequate microcirculation perfusion, affecting kidney perfusion and oxygen supply. These changes can impair kidney function and increase the risk of AKI.

This study has several limitations. Firstly, the data used in this study were obtained from the MIMIC-IV database, which only includes patients from medical institutions in the United States, and therefore may not fully represent populations from other countries or regions. Additionally, there may be selection bias among the patients included in this database, so caution is needed when extrapolating the study results. Secondly, this study used a retrospective cohort study design, which may suffer from information bias and omissions due to the pre-existing nature of the study subjects, which could potentially affect the accuracy of the results. Moreover, the definition of AKI in this study was based on changes in serum creatinine levels, rather than other biomarkers or clinical presentations. Thirdly, the sample size of patients in the subgroup who received red blood cell transfusion may be small, which could affect the reliability of statistical analyses and the feasibility of generalizing the study results. Therefore, larger-scale studies are needed to validate these findings. Fourthly, this study doesn't describe what happens during the surgery and CPB and is blinded about RBC transfusion and Hb trajectories during surgery. Furthermore, the use of Goal Directed Perfusion strategy during CPB may have a significant influence on postoperative AKI. Fifthly, the long time span (11 years) could pose several challenges. For example, changes in clinical practice, technology, or patient characteristics over time may introduce confounding factors that we cannot fully account for. Additionally, data collection methods and quality may have varied over the years, which could affect the accuracy and completeness of our results. Finally, hemoglobin elevation after RBC transfusion is harmful to the kidney, but we should be more cautious with a general relationship between hemoglobin elevation from other etiologies than transfusion (i.e., haemoconcentration, iron supply, erythropoietin stimulation) and AKI. Additionally, the threshold for RBC transfusion can be challenged by the metabolic tolerance of anemia assessed by SvO_2_ (35, 36).

## 5. Conclusion

This study has identified a trajectory of hemoglobin levels that is associated with an increased risk of postoperative AKI following cardiac surgery. It should be noted that a fixed hemoglobin threshold should not be applied to all types of patients. Among patients receiving red blood cell transfusions, maintaining hemoglobin levels above 10 g/dl through red blood cell transfusions is associated with an increased risk of AKI.

## Data Availability

The raw data supporting the conclusions of this article will be made available by the authors, without undue reservation.
