# Supplementary material for: Longitudinal hemoglobin trajectories and acute kidney injury in patients undergoing cardiac surgery: a retrospective cohort study
Source: Front Cardiovasc Med. 2023 May 17;10:1181617. doi: 10.3389/fcvm.2023.1181617 (PMC10229827; doi:10.3389/fcvm.2023.1181617)
Supplement: Supplementary file 1 [file Datasheet1.pdf]

Supplementary Material

Longitudinal Hemoglobin Trajectories and Acute Kidney Injury in Patients undergoing Cardiac Surgery: A Retrospective Cohort Study

Table S1 Trajectory Model Fitting.

Table S2 The VIFs of each covariate.

Table S3 Validation of the hemoglobin threshold in the prior literature.

Table S4 Crude comparison of trajectories of hemoglobin between groups, excluding AKI-III patients (sensitivity analysis)

Table S5 Sensitivity analysis: Excluding patients with AKI-III, investigate the association between hemoglobin levels and postoperative AKI in patients undergoing cardiopulmonary bypass surgery.

Figure. S1 Missing value in the study.

Figure. S2 Love plot of balance in baseline and clinical characteristics before and after the inverse-probability-of-treatment weighting (IPW).

Figure. S3 Trajectory plot of patients with three dynamic hemoglobin trajectory patterns after excluding patients with AKI-III (Sensitivity analysis).

Table S1 Trajectory Model Fitting.

Fit statistic	Number of classes					
	1	2	3	4	5	6
AIC	-44994.93	-42444.25	-41788.35	-41599.37	-41330.63	-41255.92
BIC	-45010.95	-42476.28	-41836.4	-41663.44	-41404.31	-41329.6
Δ BIC		2534.67	639.88	172.96	259.13	74.71
Bayes Factor (2*Δ BIC)		5069.34	1279.76	345.92	518.26	149.42
Class proportion	Class 1, 100%	Class 1, 66.9%<br>Class 2, 33.1%	Class 1, 44.5%<br>Class 2, 43.7%<br>Class 3, 11.8%	Class 1, 38.1%<br>Class 2, 4.5%<br>Class 3, 44.8%<br>Class 4, 12.6%	Class 1, 30.3%<br>Class 2, 5.9%<br>Class 3, 43.6%<br>Class 4, 18.4%<br>Class 5, 1.8%	Class 1, 28.1%<br>Class 2, 6.1%<br>Class 3, 42.7%<br>Class 4, 3.0%<br>Class 5, 19.9%<br>Class 6, 0.2%
AvePP		Class 1, 0.94<br>Class 2, 0.90	Class 1, 0.88<br>Class 2, 0.82<br>Class 3, 0.86	Class 1, 0.79<br>Class 2, 0.75<br>Class 3, 0.82<br>Class 4, 0.86	Class 1, 0.76<br>Class 2, 0.74<br>Class 3, 0.77<br>Class 4, 0.83<br>Class 5, 0.85	Class 1, 0.75<br>Class 2, 0.74<br>Class 3, 0.76<br>Class 4, 0.83<br>Class 5, 0.79<br>Class 6, 0.93

AIC: Akaike’s information criterion, BIC: Bayesian information criteria, AvePP: average posterior probabilities.

Table S2 The VIFs of each covariate.

	Original cohort (n=4478)			RBC transfusion subgroup (n=1939)			Non-RBC transfusion subgroup (n=2539)		
	GVIF	Df	GVIF^(1/(2*Df))	GVIF	Df	GVIF^(1/(2*Df))	GVIF	Df	GVIF^(1/(2*Df))
Traj_Group	1.181	2	1.043	1.133	2	1.032	1.249	2	1.057
Gender	1.235	1	1.111	1.180	1	1.086	1.284	1	1.133
Age	1.189	1	1.091	1.162	1	1.078	1.193	1	1.092
Initial_Weight	1.281	1	1.132	1.270	1	1.127	1.293	1	1.137
Emergency	1.405	1	1.185	1.384	1	1.176	1.510	1	1.229
Diabetes	1.290	1	1.136	1.290	1	1.136	1.318	1	1.148
Hypertension	1.060	1	1.030	1.062	1	1.031	1.068	1	1.033
HF	1.114	1	1.055	1.102	1	1.050	1.175	1	1.084
CHD	3.123	1	1.767	2.957	1	1.719	3.251	1	1.803
Initial_WBC	1.049	1	1.024	1.053	1	1.026	1.055	1	1.027
Sodium	1.172	1	1.083	1.138	1	1.067	1.301	1	1.141
Potassium	1.174	1	1.083	1.184	1	1.088	1.186	1	1.089
Calcium	1.104	1	1.051	1.091	1	1.044	1.174	1	1.084
Lactate	1.214	1	1.102	1.241	1	1.114	1.220	1	1.105
Glucose	1.398	1	1.182	1.365	1	1.168	1.490	1	1.221
Vasopressoruse	1.037	1	1.018	1.025	1	1.012	1.070	1	1.034
Coronary_angiography	1.237	1	1.112	1.234	1	1.111	1.274	1	1.129
Operation	3.590	3	1.237	3.356	3	1.224	3.814	3	1.250

Note: GVIF= generalized variance inflation factors. Df= degree of freedom.

Table S3 Validation of the hemoglobin threshold in the prior literature.

	Original cohort (n=4478)			RBC transfusion subgroup (n=1939)			Non-RBC transfusion subgroup (n=2539)		
	8 g/dL	10 g/dL	12 g/dL	8 g/dL	10 g/dL	12 g/dL	8 g/dL	10 g/dL	12 g/dL
AUC	0.552	0.545	0.529	0.508	0.517	0.506	0.531	0.526	0.506
Sensitivity	0.25	0.63	0.91	0.30	0.30	0.96	0.12	0.48	0.81
Specificity	0.86	0.46	0.15	0.71	0.73	0.06	0.94	0.57	0.20
PPV	0.27	0.20	0.19	0.30	0.31	0.30	0.18	0.10	0.10
NPV	0.84	0.85	0.89	0.72	0.72	0.76	0.91	0.91	0.91
PLR	1.71	1.17	1.07	1.05	1.12	1.01	2.05	1.12	1.02
NLR	0.88	0.80	0.60	0.98	0.95	0.79	0.93	0.91	0.94

Abbreviations: ICP = intracranial pressure; AUC = area under the curve; PPV = positive predictive value

NPV = negative predictive value; PLR = positive likelihood ratio; NLR = negative likelihood ratio

Table S4 Crude comparison of trajectories of hemoglobin between groups, excluding AKI-III patients (sensitivity analysis)

	Traj-1 group (N=1855)	Traj-2 group (N=1964)	Traj-3 group (N=571)	P
Initial Hb (g/dL)	8.50 [7.70;9.40]	10.3 [9.60;11.3]	12.2 [11.3;13.4]	0
Maximal Hb (g/dL)	10.5 [9.90;11.1]	11.9 [11.3;12.5]	13.6 [13.0;14.2]	0
Initial platelet (109/L)	179 [132;240]	174 [135;225]	175 [139;227]	0.296
Minimal platelet (109/L)	113 [89.0;145]	119 [97.8;148]	124 [100;153]	<0.001
Initial creat (mg/dL)	0.90 [0.80;1.20]	0.90 [0.80;1.10]	0.90 [0.80;1.10]	0.018
Maximal creat (mg/dL)	1.10 [0.90;1.50]	1.00 [0.90;1.20]	1.00 [0.90;1.20]	<0.001
RBC infusion, n (%)	1266 (68.2%)	572 (29.1%)	29 (5.08%)	<0.001
Operation, n (%)				<0.001
Coronary artery bypass grafting	847 (45.7%)	1115 (56.8%)	359 (62.9%)	
Operation on valves	438 (23.6%)	362 (18.4%)	94 (16.5%)	
Coronary bypass with valves	327 (17.6%)	236 (12.0%)	42 (7.36%)	
Other	243 (13.1%)	251 (12.8%)	76 (13.3%)	
Vasopressor use, n (%)	1611 (86.8%)	1622 (82.6%)	397 (69.5%)	<0.001
Coronary angiography, n (%)	582 (31.4%)	597 (30.4%)	232 (40.6%)	<0.001
Los hospital (day, median [IQR])	7.88 [5.33;11.2]	6.28 [5.01;8.86]	6.91 [5.11;10.1]	<0.001
Los icu (day, median [IQR])	2.16 [1.29;3.41]	1.45 [1.19;2.86]	1.38 [1.15;2.50]	<0.001
Initial sofa (scores, median [IQR])	3.00 [1.00;5.00]	2.00 [1.00;4.00]	2.00 [0.00;4.00]	<0.001
Maximal sofa (scores, median [IQR])	6.00 [4.00;8.00]	5.00 [4.00;7.00]	5.00 [3.00;7.00]	<0.001
Cardiac output (L/min, median [IQR])	4.11 [3.30;5.10]	4.57 [3.60;5.53]	5.26 [4.18;6.01]	<0.001
Acute posthemorrhagic anemia, n (%)	364 (19.6%)	224 (11.4%)	53 (9.28%)	<0.001
In-hospital death, n (%)	24 (1.29%)	10 (0.51%)	4 (0.70%)	0.032
AKI Stage, n (%)				<0.001
I	321 (17.3%)	177 (9.01%)	39 (6.83%)	
II	107 (5.77%)	48 (2.44%)	14 (2.45%)	

AKI: acute kidney injury; ICU: intensive care unit; Hb: hemoglobin; IQR: interquartile range; LOS: length of stay; RBC: red blood cell; SOFA: Sequential Organ Failure Assessment.

* Vasopressor use including dobutamine, dopamine, epinephrine, norepinephrine phenylephrine and asopressin.

# Data on cardiac output represented in the present study include only participants with no missing data.

Table S5 Sensitivity analysis: Excluding patients with AKI-III, investigate the association between hemoglobin levels and postoperative AKI in patients undergoing cardiopulmonary bypass surgery.

Model	Cluster	Original cohort		
		OR	95%CI	P
Unadjusted Model	Traj-1		Reference	
	Traj-2	0.43	(0.36-0.51)	<0.001
	Traj-3	0.34	(0.25-0.46)	<0.001
$Model_1$	Traj-1			
	Traj-2	0.56	(0.47-0.67)	<0.001
	Traj-3	0.55	(0.40-0.76)	<0.001
$Model_2$	Traj-1			
	Traj-2	0.59	(0.49-0.71)	<0.001
	Traj-3	0.57	(0.41-0.78)	<0.001
$Model_3$	Traj-1			
	Traj-2	0.59	(0.49-0.71)	<0.001
	Traj-3	0.55	(0.39-0.75)	<0.001
$Model_4$	Traj-1			
	Traj-2	0.62	(0.51-0.75)	<0.001
	Traj-3	0.62	(0.44-0.86)	0.005
$Model_{IPW}$	Traj-1			
	Traj-2	0.57	(0.48-0.68)	<0.001
	Traj-3	0.70	(0.54-0.89)	0.007

AKI: acute kidney injury; RBC: red blood cell. IPW: inverse probability of treatment weighting.

$Model_1$: adjusted for age, gender, initial weight and emergency.

$Model_2$: additionally adjusted for diabetes, hypertension, hear failure and coronary hear disease upon $Model_1$.

$Model_3$: additionally adjusted for initial white blood cells, serum sodium, serum potassium, serum calcium, serum lactate and serum glucose upon $Model_2$.

$Model_4$: additionally adjusted for vasopressor use, coronary angiography and operation upon $Model_3$.

$Model_{IPW}$: adjusted for all aforementioned covariates using the IPW method.

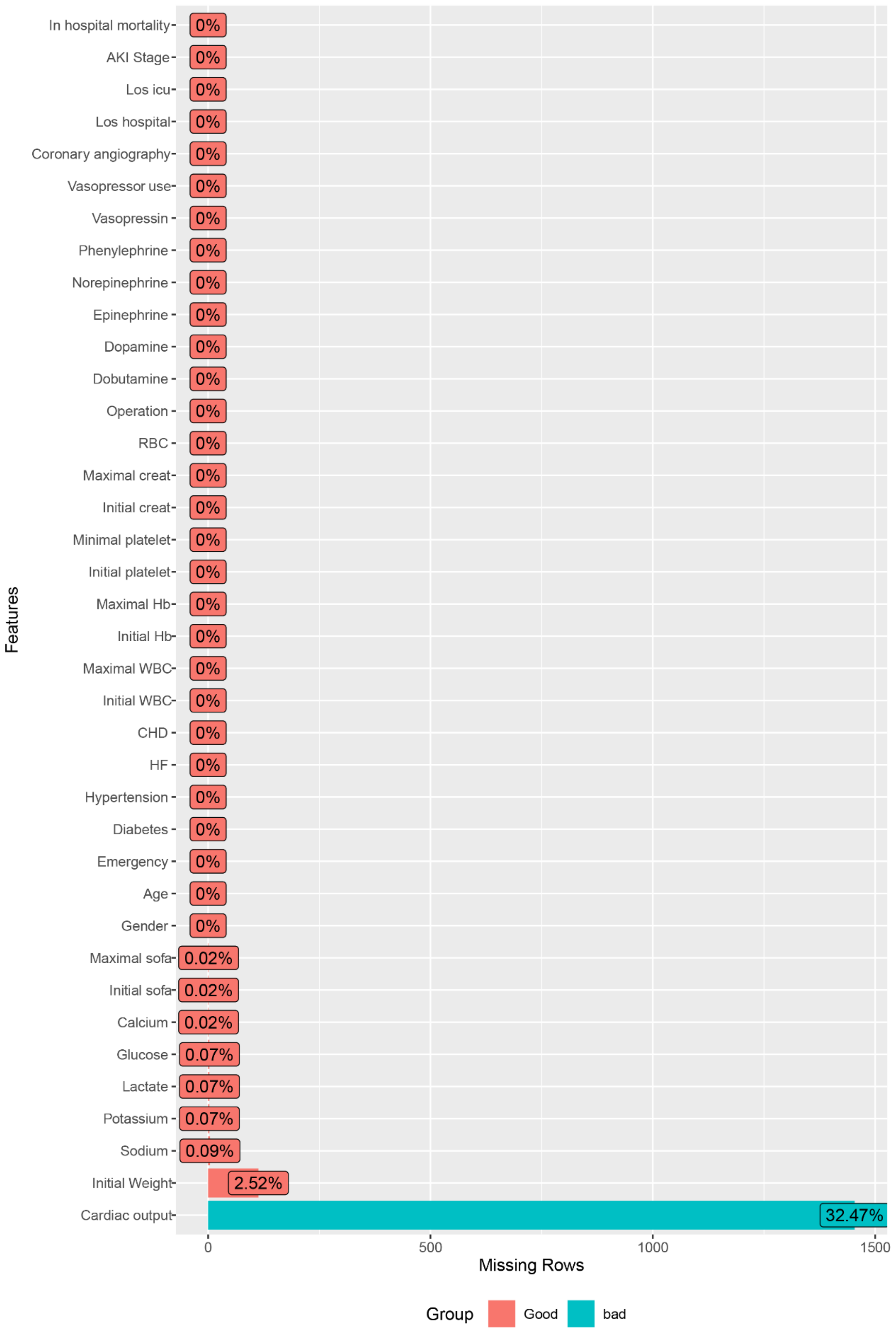

Figure. S1 Missing value in the study.

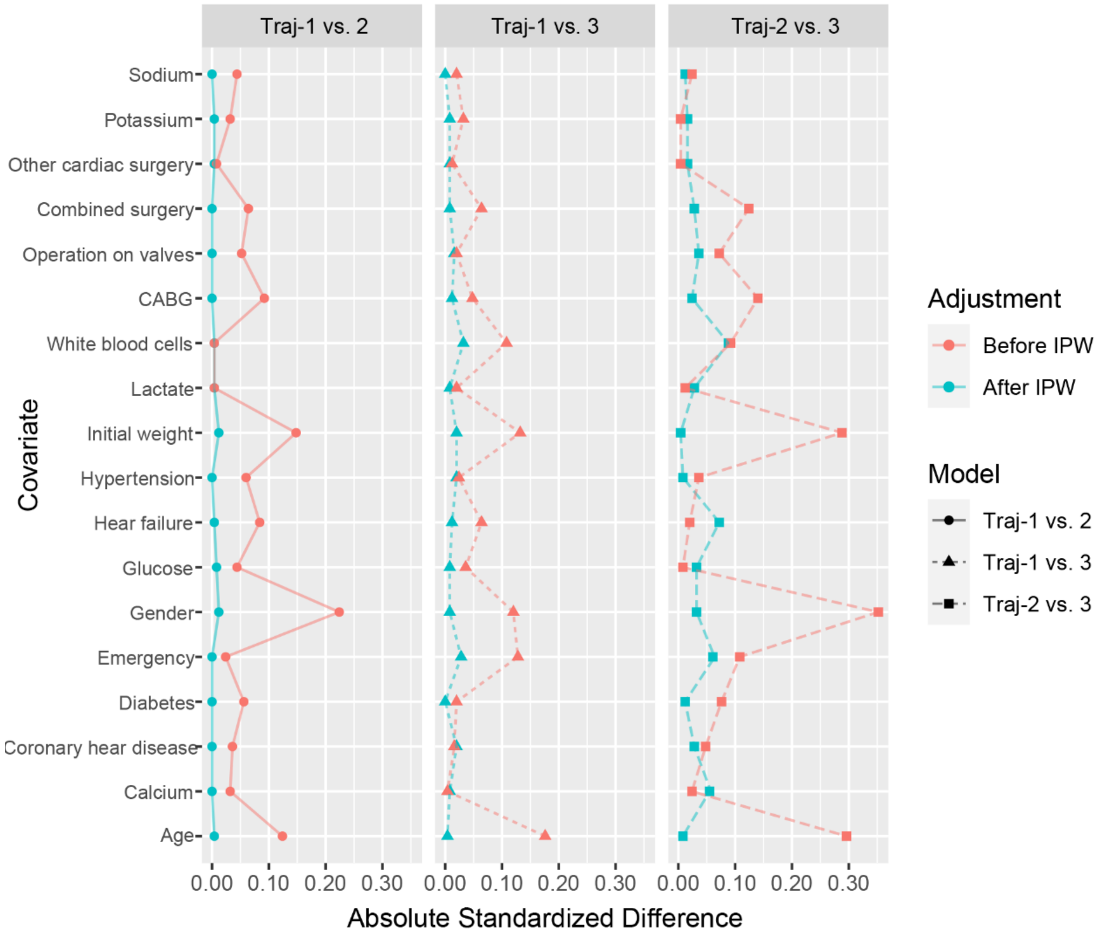

Figure. S2 Love plot of balance in baseline and clinical characteristics before and after the inverse-probability-of-treatment weighting (IPW).

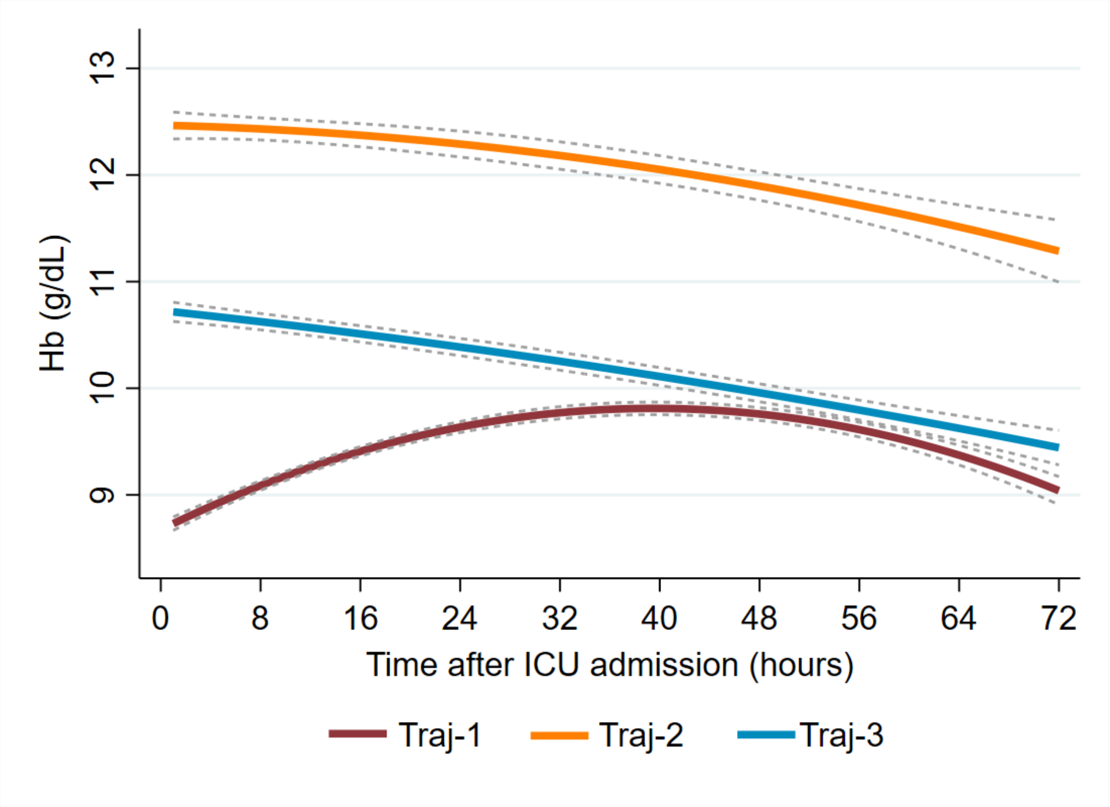

Figure. S3 Trajectory plot of patients with three dynamic hemoglobin trajectory patterns after excluding patients with AKI-III (Sensitivity analysis).
